# Genetic susceptibility modifies the association of long-term air pollution exposure on Parkinson’s disease

**DOI:** 10.1038/s41531-024-00633-1

**Published:** 2024-01-17

**Authors:** Yi-Ming Huang, Ya-Hui Ma, Pei-Yang Gao, Xi-Han Cui, Jia-Hui Hou, Hao-Chen Chi, Yan Fu, Zhi-Bo Wang, Jian-Feng Feng, Wei Cheng, Lan Tan, Jin-Tai Yu

**Affiliations:** 1 Department of Neurology, Qingdao Municipal Hospital, Qingdao University, Qingdao, China; 2 Department of Neurology, The Affiliated Hospital of Qingdao University, Qingdao, China; 3 Department of Neurology and National Center for Neurological Disorders, Huashan Hospital, State Key Laboratory of Medical Neurobiology and MOE Frontiers Center for Brain Science, Shanghai Medical College, Fudan University, Shanghai, 200040 China; 4 Department of Neurology, Xuanwu Hospital, Capital Medical University, Changchun Street 45, Beijing, China; 5 Innovation Center for Neurological Disorders and Department of Neurology, Xuanwu Hospital, Capital Medical University, National Center for Neurological Disorders, Beijing, China; 6 Institute of Science and Technology for Brain-Inspired Intelligence, Fudan University, Shanghai, 200433 China; 7 Key Laboratory of Computational Neuroscience and Brain-Inspired Intelligence (Fudan University), Ministry of Education, Shanghai, 200040 China; 8 Fudan ISTBI—ZJNU Algorithm Centre for Brain-Inspired Intelligence, Zhejiang Normal University, Jinhua, 321004 China; 9 MOE Frontiers Center for Brain Science, Fudan University, Shanghai, 200032 China; 10 Zhangjiang Fudan International Innovation Center, Shanghai, 200433 China

**Keywords:** Parkinson's disease, Risk factors

## Abstract

Inconsistent findings exist regarding the potential association between polluted air and Parkinson’s disease (PD), with unclear insights into the role of inherited sensitivity. This study sought to explore the potential link between various air pollutants and PD risk, investigating whether genetic susceptibility modulates these associations. The population-based study involved 312,009 initially PD-free participants with complete genotyping data. Annual mean concentrations of PM_2.5_, PM_10_, NO_2_, and NO_x_ were estimated, and a polygenic risk score (PRS) was computed to assess individual genetic risks for PD. Cox proportional risk models were employed to calculate hazard ratios (HR) and 95% confidence intervals (CI) for the associations between ambient air pollutants, genetic risk, and incident PD. Over a median 12.07-year follow-up, 2356 PD cases (0.76%) were observed. Compared to the lowest quartile of air pollution, the highest quartiles of NO_2_ and PM_10_ pollution showed HRs and 95% CIs of 1.247 (1.089–1.427) and 1.201 (1.052–1.373) for PD incidence, respectively. Each 10 μg/m^3^ increase in NO_2_ and PM_10_ yielded elevated HRs and 95% CIs for PD of 1.089 (1.026–1.155) and 1.363 (1.043–1.782), respectively. Individuals with significant genetic and PM_10_ exposure risks had the highest PD development risk (HR: 2.748, 95% CI: 2.145–3.520). Similarly, those with substantial genetic and NO_2_ exposure risks were over twice as likely to develop PD compared to minimal-risk counterparts (HR: 2.414, 95% CI: 1.912–3.048). Findings suggest that exposure to air contaminants heightens PD risk, particularly in individuals genetically predisposed to high susceptibility.

## Introduction

With a global impact on over 6 million individuals, Parkinson’s disease (PD) stands as the second most prevalent neurodegenerative disorder^1^. Unfortunately, its incidence has significantly increased over the last three decades, with a 2.5-fold rise^2^. PD has prominent clinical manifestations, including resting tremor, bradykinesia, rigidity, and postural instability. Such indicators have a significant effect on the quality of life of PD patients. Given the progressive and irreversible nature of this disease, the prevention of PD is of paramount importance. However, despite extensive research, PD’s underlying risk factors remain poorly understood. It highlights the urgent need for further investigation and intervention to improve our understanding and develop effective strategies for prevention.

Various environmental factors have been considered potential triggers for PD development for many years. According to the Braak hypothesis, alpha-synuclein’s pathological aggregation commences in the gastrointestinal tract and olfactory bulb prior to extending toward the central nervous system^3^. Consequently, exposure to environmental contaminants, including pesticides, metals, and microorganisms, has been suggested to potentially contribute to the early pathology of PD and ultimately increase the risk of developing the disease^4^. Despite air pollution still being the most prevailing environment-related risk to the health of human beings, the findings of epidemiological research looking into the association between the said pollution type and PD are varied^5–15^. While recent studies have suggested a potential association between long-term exposure to air pollution and the onset of PD, the significance of this relationship remains inconclusive due to limitations such as small sample sizes^7,10^, a low number of cases^6^, short exposure or follow-up durations^6,7,9,11,12,14,15^, and a lack of adjustments for other exposure factors, such as smoking^5,15,16^. Therefore, there is a need for more extensive population-based cohort research to investigate this possible link thoroughly.

In addition to environmental exposures, genetic susceptibility has been demonstrated as another significant risk factor for PD^17^. Recent genome-wide association studies (GWAS) have uncovered numerous genetic variants associated with an increased PD risk^18,19^. In this context, formulating polygenic risk scores (PRS) by aggregating multiple variants demonstrates the capacity to identify susceptible individuals^20^. The occurrence and progression of PD are influenced by both genetic and environmental factors^20,21^, with the modifying role of genetic susceptibility in the relationship between air pollution and PD risk still largely unknown. In this context, we formulated specific hypotheses to guide our investigation. First, we hypothesized that prolonged exposure to various air pollutants (i.e., PM_2.5_, PM_10_, NO_2_, and NO_x_) is positively correlated with the incidence of PD. Second, we postulated that there are synergistic effects and interactions between genetic factors and air pollutant exposure, potentially modifying the overall risk of PD.

## Results

### Descriptive results

Over 2,685,412 person-years and 2356 incident PD events were recorded (median follow-up: 12.07 years, range: 10.46–15.01 years). Table 1 provides the baseline properties of the research subjects. Those who developed PD had a greater probability of being elderly, less affluent, with higher BMI, with a greater risk genetics-wise, and with exposure to more pollutants in the air. Pearson correlation results and summarized statistics for air pollutants are displayed in Supplementary Table 1.

**Table 1:** Descriptive characteristics of the study participants.

Variables	Total (*n* = 312,009)	Individuals without PD (*n* = 309,653)	Individuals with PD (*n* = 2,356)	*P* values
Age, mean (SD), *y*	57.01 (8.01)	57.27 (7.91)	63.18 (5.14)	<0.001
Sex, *n* (%)				
Male	145,481(47)	143,988 (46)	1493(63)	<0.001
Female	166,528 (53)	165,665(54)	863 (37)	<0.001
Education level, *n* (%)				0.004
College or university degree	91,618 (29)	90,989 (29)	629 (27)	
Other	220,391 (69)	218,664 (71)	1727 (73)	
Employment status, *n* (%)				0.130
Employed or retired	289,120(93)	284,516 (92.6)	2195 (93.6)	
Others	20,480 (6.6)	20,343 (6.6)	137 (5.8)	
Unknown	2409	2397	12	
Household income, *n* (%)				<0.001
<£18,000	62,595 (24)	61,907 (23)	688 (35)	
£18,000–29,999	70,595 (26)	70,006 (26)	589 (30)	
£30,000–51,999	69,586 (26)	69,183 (26)	403 (21)	
£52,000–100,000	51,581 (19)	51,373 (19)	208 (11)	
>£100,000	12,658 (4.9)	12.601 (4.8)	57 (2.9)	
Unknown	44,994	44,583	411	
Townsend deprivation index, mean (SD)	−1.57 (2.89)	−1.57 (2.89)	−1.53 (2.99)	0.900
BMI, mean (SD)	27.6 (4.8)	27.5 (4.8)	27.8 (4.7)	0.003
Alcohol, *n* (%)				<0.001
Never	9997 (4.5)	9885 (3.2)	112 (4.8)	
Previous	10,820 (3.6)	10,685 (3.5)	135 (5.7)	
Current	290,918 (92)	288,816 (93)	2102 (89)	
Unknown	274	267	7	
Smoking, *n* (%)				<0.001
Never	166,451 (54)	165,242 (54)	1209 (52)	
Previous	112,299 (35)	111,319 (36)	980 (42)	
Current	32,120 (11)	31,965 (10)	155 (6.6)	
Unknown	1139	1127	12	
No. of long-term morbidities				<0.001
None	109,008 (35)	108,465 (35)	543 (23)	
1	99,994 (32)	99,305 (32)	689 (29)	
2	57,170 (18)	56,633 (18)	537 (23)	
3	26,775 (8.6)	26,453 (8.5)	322 (14)	
4	11,259 (3.6)	11,118 (3.6)	141 (6.0)	
≥5	7803 (2.5)	7679 (2.5)	124 (5.3)	
Genetic risk category, *n* (%)				<0.001
Low	104,002 (33)	103,466 (33)	536 (23)	
Intermediate	104,004 (33)	103,275 (33)	729 (31)	
High	104,003 (33)	102,912 (33)	1091 (46)	
Air pollution, μg/m^3^, mean (SD)				
PM_2.5_	9.94 (1.04)	9.94 (1.04)	9.93 (1.03)	<0.001
PM_10_	19.08 (1.83)	19.07 (1.83)	19.12 (1.81)	<0.001
NO_2_	28.14 (8.38)	28.14 (8.38)	28.43 (8.51)	<0.001
NO_x_	42.97 (14.87)	42.97 (14.88)	43.00 (14.48)	<0.001

Continuous variables are displayed as means ± SD, and categorical variables are displayed as numbers (percentages).

### Association between different air pollutants and subsequent Parkinson’s disease

We examined the link between significant air pollutants and PD risk. In the final multivariable-adjusted model, we found that participants exposed to the highest NO_2_ quartile had a markedly increased PD risk than those exposed to the lowest quartile, with HRs of 1.247 and 95% CI between 1.089 and 1.427. Equally important, subjects exposed to the highest quartile of PM_10_ showed a 20.1% increased risk of PD compared to subjects in the lowest quartile (HRs = 1.201; 95% CI: 1.052–1.373). On the other hand, our study found no significant correlations between other air pollutants (e.g., PM_2.5_ and NO_x_) and the incidence of PD (Table 2). The risk of PD increased by 8.9% for every 10 μg/m^3^ rise in NO_2_ exposure (HR: 1.089; 95% CI: 1.026–1.155) and by 36.3% for every 10 μg/m^3^ rise in PM_10_ exposure (HR: 1.363; 95% CI: 1.043–1.782). The sensitivity analyses did not significantly alter these results (Supplementary Tables 2–4). As depicted in Supplementary Fig. 1, we investigated the dose-response relationship between each air pollutant and PD events, and NO_2_ showed a relatively strong effect (nonlinear *P* = 0.007). Moreover, we conducted additional stratified analyses and observed a robust correlation between PM_10_ and NO_2_ exposure and the risk of developing PD in the population under 65 but not in those aged 65 or older. In a similar fashion, PM_10_ and NO_2_ were found to have significance alongside PD risk in males but not in females. Notably, significant correlations between air pollution and PD risk were also evident within populations with a higher Townsend deprivation index and an increased count of long-term morbidities (Supplementary Table 5).

**Table 2:** Association between long-term exposure to air pollutants and Parkinson’s Disease (PD).

Air pollution	No. PD cases/person-years	PD HR (95% CI)		
		Model 1^a^	Model 2^b^	Model 3^c^
NO_2_				
Q1	568/594,834	1.000 (Reference)	1.000 (Reference)	1.000 (Reference)
Q2	558/602,460	0.954 (0.849–1.072)	0.981 (0.861–1.119)	0.981 (0.861–1.118)
Q3	587/604,680	1.002 (0.892–1.124)	1.062 (0.932–1.210)	1.064 (0.934–1.213)
Q4	643/589,576	1.104 (0.986–1.236)	1.243 (1.086–1.423)	1.247 (1.089–1.427)
per 10-μg/m^3^ increase		1.038 (0.989–1.089)	1.085 (1.022–1.151)	1.089 (1.026–1.155)
* P* for trend		0.053	<0.001	<0.001
NO_X_				
Q1	608/594,734	1.000 (Reference)	1.000 (Reference)	1.000 (Reference)
Q2	573/597,884	0.939 (0.838–1.052)	0.976 (0.858–1.109)	0.974 (0.856–1.107)
Q3	571/698,694	0.942 (0.841–1.056)	1.028 (0.903–1.170)	1.028 (0.903–1.171)
Q4	604/600,237	0.945 (0.845–1.058)	1.052 (0.918–1.206)	1.055 (0.920–1.209)
per 20-μg/m^3^ increase		0.978 (0.926–1.033)	1.019 (0.955–1.086)	1.020 (0.956–1.087)
* P* for trend		0.367	0.363	0.341
PM_2.5_				
Q1	592/589,938	1.000 (Reference)	1.000 (Reference)	1.000 (Reference)
Q2	580/598,782	0.965 (0.860–1.082)	1.001 (0.882–1.138)	1.001 (0.881–1.137)
Q3	594/600,146	0.975 (0.870–1.093)	1.007 (0.885–1.147)	1.007 (0.885–1.147)
Q4	590/602,683	0.927 (0.827–1.039)	0.998 (0.871–1.144)	0.998 (0.871–1.145)
per 5-μg/m^3^ increase		0.846 (0.696–1.027)	0.904 (0.713–1.145)	0.903 (0.713–1.145)
* P* for trend		0.24	0.997	0.992
PM_10_		1.123 (0.894–1.410)	1.365 (1.061–1.756)	1.359 (1.055–1.751)
Q1	552/593,853	1.000 (Reference)	1.000 (Reference)	1.000 (Reference)
Q2	606/598,914	1.059 (0.943–1.188)	1.094 (0.961–1.245)	1.094 (0.961–1.245)
Q3	583/605,131	0.947 (0.843–1.064)	0.996 (0.873–1.136)	1.000 (0.877–1.140)
Q4	615/593,652	1.066 (0.950–1.196)	1.194 (1.045–1.364)	1.201 (1.052–1.373)
per 10-μg/m^3^ increase		1.123 (0.894–1.410)	1.340 (1.025–1.752)	1.363 (1.043–1.782)
* P* for trend		0.670	0.047	0.036

*NO*_*2*_ nitrogen dioxide, *NO*_*X*_ nitrogen oxides, *PM*_*2.5*_ particulate matter 2.5, *PM*_*10*_ particulate matter 10, *HR* hazard ratio, *CI* confidence interval.

^a^Model 1, Unadjusted.

^b^Model 2, Adjusted for age, sex, BMI, alcohol consumption, smoking status, education level, employment status, household income, and Townsend deprivation index.

^c^Model 3, Included model 2 plus the number of long-term morbidities.

### Joint effect of PD-PRS and air pollution on Parkinson’s disease risk

**Fig. 1:**
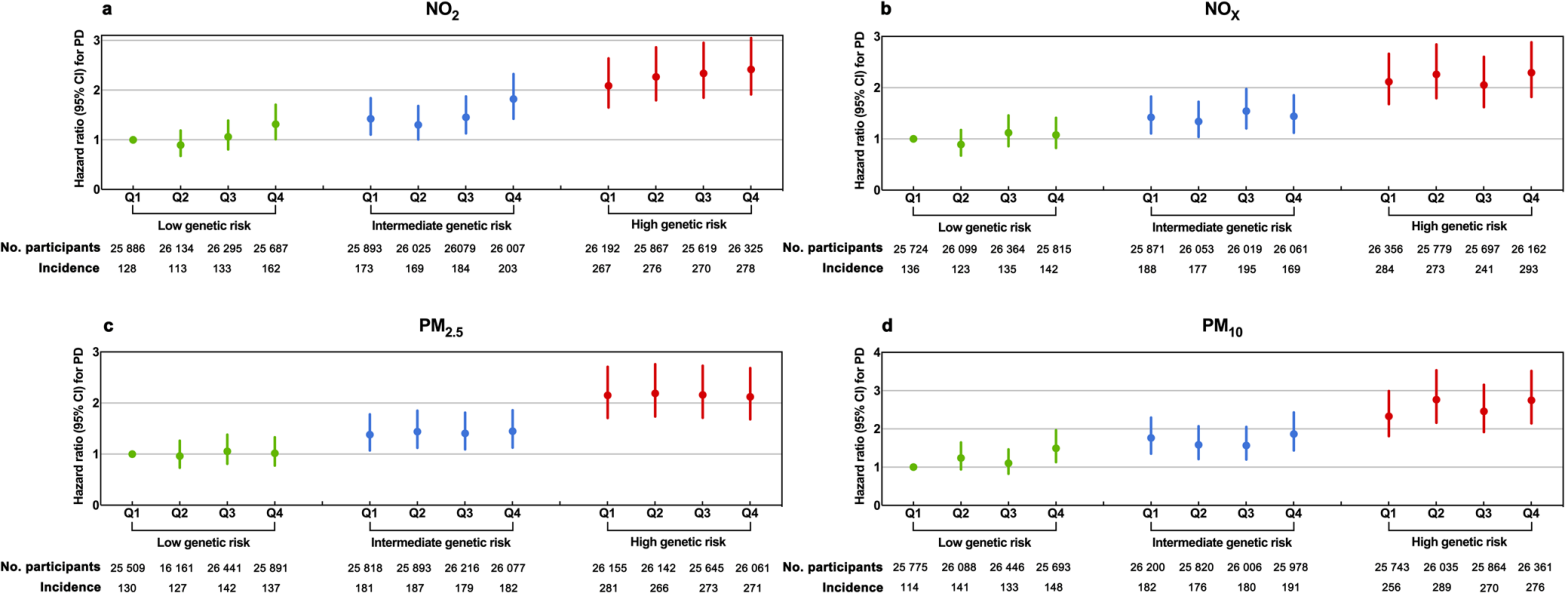
Associations of air pollution and genetic risk with incident PD. The joint associations of long-term NO_2_ (**a**), NO_x_ (**b**), PM_2.5_ (**c**), and PM_10_ (**d**) exposure and PRS with the risk of incident PD. Cox regression models adjusted for age, sex, BMI, alcohol status, smoking status, education level, employment status, household income, Townsend deprivation index, genotyping array, the number of long-term morbidities, and the first 10 principal components of ancestry. Error bars represent HR with 95% confidence intervals (95% CI).

Supplementary Table 6 in the Supplementary Information overviews the PRS’s association with PD risk. A multivariable-adjusted model established a statistically significant link between the PRS and PD (HR: 1.482, 95% CI: 1.352–1.623). Moreover, the high genetic risk group exhibited a substantially elevated incidence of PD, with an HR (95% CI) of 2.182 (1.952–2.438) compared to the low genetic risk group. These findings retained their stability across various multivariate-adjusted models. Figure 1 depicts the risk of developing PD concerning combined exposure to air pollutants and genetic risk. Individuals with elevated air pollutant exposure levels and heightened genetic risk were found to face an increased risk of developing PD [PM_10_: 2.748 (2.145–3.520), NO_2_: 2.414 (1.912–3.048), NO_x_: 2.294 (1.822–2.890)], in contrast to those with low air pollutant exposure levels and minimal genetic risk. Significant interactions between the PRS and NO_2_, NO_x_, PM_2.5_, and PM_10_ were detected (all P-_Multiplicative interactions_ < 0.001), underscoring their synergistic effects.

## Discussion

Within this research endeavor, we scrutinized the correlation between air pollutants and the risk of developing PD while factoring in genetic susceptibility. Our investigation unveiled a noteworthy connection between overall air pollution levels and an escalated risk of PD onset. Notably, persistent exposure to NO_2_ and PM_10_ emerged as autonomous risk factors for the subsequent manifestation of PD. Upon evaluating the compounded effects of genetic predisposition and air pollutant exposure, we detected a considerable amplification in the risk of PD among participants possessing heightened genetic susceptibility and concurrently exposed to elevated air pollutant concentrations. Furthermore, we identified a substantial association between genetic-based sensitivity and exposure to air pollution. The consequences of protracted air pollution on PD may be exacerbated by genetic susceptibility.

Little is known about the relationship between exposure to air pollution and PD development. The link between long-term air pollutant exposure and PD incidence was extensively examined. According to previous research, we calculated long-term exposures using data on air pollution from 2010. The levels of air pollution in the United Kingdom are still quite stabilized, and the majority of temporal air pollution tendencies are commonly stable throughout the duration covered by the research. Hence, mean air pollution may serve as a surrogate for long-term exposure. Using average levels of air pollution as an analysis estimate further demonstrates the validity of this method. In prospective research involving 78,830 Korean participants, scholars discovered a 42% increased PD risk among subjects exposed to the highest NO_2_ quartile versus the lowest quartile^6^. Our findings are consistent with these findings. Notably, that study did not find a correlation between PM_2.5_ exposure and PD incidence. Similarly, Canadian prospective cohort research detected a correlation between NO_2_ exposure and the incidence of PD^15^. Our findings, however, contradict those of various other investigations. Zhu et al. determined that exposure to ambient PM_2.5_, but not NO_2_ or PM_10_, for the long term, was related to PD development^22^. These inconsistencies may be attributed to differences in study design and other factors, such as the number of PD outcomes, including or excluding other gaseous pollutants, and variations in the constituent factors in PM across different regions. Our study contributes new evidence to the epidemiological study of PD-associated atmospheric pollution. Our findings have consequences for policies and clinical tests, as variations in policies and personal behavior may aid in mitigating air pollution, which may lead to a decrease in PD.

Several potential mechanisms could help explain the connection between air pollution and PD. One of these mechanisms involves the induction of neuroinflammation and oxidative stress by air pollutants, which can promote the onset and development of PD^23,24^. Air pollutants can enter the circulatory system through penetration of the olfactory mucosa or interstitial lung tissue compartments, resulting in the central nervous system’s inflammation and oxidative stress^25,26^. Moreover, air pollution may compromise the blood-brain barrier’s integrity, increasing its permeability to toxic substances entering circulation^27^. Notably, a mouse experiment found that air pollution increased the expression of specific inflammatory genes in the olfactory bulb, a brain region where PD pathology commonly appears early^28–30^. An increasing body of research indicates that environmental toxins entering the gastrointestinal tract may cause the formation of pathological alpha-syn in the gut, which can then spread to the central nervous system and cause the loss of dopaminergic neurons^31,32^. However, direct evidence supporting this theory is scarce. Thus, additional research is required to ascertain the precise mechanisms underlying the pathogenesis of PD due to air pollution.

For this research, we investigated the link between air pollutants and the incidence of PD in the UK Biobank using PRS. Our results signify that increased air pollution and PD-PRS are related to an increased PD risk, with a substantial interaction between air pollution and PRS categories observed in both joint and stratified analyses. It implies a gene-environment interaction in the occurrence of PD. In addition, individuals with lower levels of air pollution had a significantly lower risk of PD occurrence across all PRS tertiles. Therefore, our findings imply that reducing air pollution levels would provide a potential benefit for individuals who are genetically predisposed to PD.

Our study has multiple strengths. To the best of our knowledge, our investigation represents the initial exploration of the impact of genetic susceptibility on the correlation between exposure to air pollution and the likelihood of developing PD. Our outcomes are more credible due to the magnitude of our sample and the length of our follow-up. In addition, the data provided by the UK Biobank enabled us to consider many confounding factors, mainly genetic susceptibility to PD. Nevertheless, our study has some limitations. As one limitation, certain risk factors for PD, including exposure to 1-methyl-4-phenyl-1,2,3,6-tetrahydropyridine and other neurotoxins that may result in motor and non-motor PD symptoms, could be excluded in the statistical modeling due to insufficient pertinent data from the UK Biobank. Another limitation is that the spectrum of air pollutants was insufficient to detect exposure to carbon monoxide, sulfur dioxide, and ozone. Moving forward, other air contaminants may need to be incorporated. Thirdly, exposure evaluation according to a specific address did not rule out exposure misclassification when considering outdoor tasks. Additional research with more precise evaluations is vital to corroborate our outcomes. Fourthly, we must recognize that covariate influence cannot be ruled out, singular contaminant associations may not exhibit independence, and our findings must be cautiously interpreted. Fifth, future global genomic studies may identify additional variants associated with PD, and incorporating more SNPs into future research may enhance genetic risk evaluation. Even though we controlled for many possible confounders in the analyses, unmeasured or unknown variables can still be residual confounders. Additionally, this study differentiated individuals into low, medium, and high-risk groups based on PRS calculated from multiple SNP loci associated with Parkinson’s disease. While this method is commonly employed in previous literature^33–35^, it has limitations as it does not distinguish populations based on specific risk loci. Future research could consider stratifying populations using information from specific loci to validate the findings more comprehensively. Furthermore, since the majority of the subjects in our study were of European descent, our results concerning the association between exposure to air pollution, genetic sensitivity to cataracts, and other populations should be interpreted with caution.

Our study leveraged prospective data to confirm the detrimental, chronic impact of PM_10_ and NO_2_ on PD development. Significantly, we discovered that individuals with elevated genetic susceptibility and heightened exposure to air pollutants faced a markedly heightened risk of PD. Therefore, applying effective techniques for mitigating air pollution as soon as possible is crucial in order to protect individuals, particularly those having a high genetic sensitivity, from a higher risk of PD development.

## Methods

### Study population

The UK Biobank provided the data for this prospective cohort research. The design and survey methodologies of the investigation have already been discussed^36^. Between 13 March 2006 and 01 October 2010, the baseline survey was administered at 22 testing centres in urbanized regions of Wales, Scotland, and England^36^. Above half a million subjects gave extensive demographic, socioeconomic, and health data via touchscreen-based surveys and anthropometric assessments^37^. The NorthWest Multi-center Research Ethics Committee (REC reference 11/NW/0382) endorsed the UK Biobank study, and prior to participation, informed consent was obtained in written form.

This investigation precluded (*N* = 64,940) persons with a history of baseline PD and those with confirmed PD based on medical data. Therefore, participants with incomplete genetic information (*N* = 48,778) and air pollution information (*N* = 34,263) were excluded. Additionally, we excluded individuals of non-White descent (self-reported by participants; *N* = 39,906), as the genetic instrument used in this study was constructed based on data from White participants. Ultimately, data from 312,009 individuals was available for the final analysis (Supplementary Fig. 2). All study participants gave informed consent in written form to guarantee participation.

### Outcomes

We used the algorithm recommended by the UK Biobank to identify participants with PD^38^. Disease information was extracted from electronic health records of hospital admissions and death registers, accessed through the Hospital Episode Statistics England, Scottish Morbidity Records, or Welsh Patient Case Databases. Following the exclusion of individuals with PD at baseline, PD occurrences were identified by analyzing admission data during subsequent follow-up visits, utilizing the International Classification of Diseases, Ninth Revision (ICD-9), or Tenth Revision (ICD-10) codes. Follow-up time was computed from baseline up to the earliest of the following: PD diagnosis, mortality, stoppage of follow-up, or censorship. Supplementary Table 7 provides data on the codes used for identifying PD cases in this research.

### Estimation of air pollutants

The UK Biobank diligently collected estimates of ambient air pollutants, including PM_2.5_, PM_10_, NO_2_, and NO_x_, spanning 2005 to 2007 and specifically focusing on 2010. During 2005–2007, comprehensive data on ambient air pollution were acquired from European Union-wide maps featuring a precise resolution of 100 × 100 meters. Systematically, the UK Biobank associated each participant’s residential address with these maps, extracting detailed air pollution concentrations within 100 × 100-meter grid cells. Moving forward to 2010, the annual average air pollution concentration was intricately calculated using an advanced land-use regression (LUR) model^39,40^. This model seamlessly integrated residential addresses collected during participants’ baseline visits and monitoring data meticulously gathered from the European Study of Cohorts for Air Pollution Effects (ESCAPE) from January 26, 2010, to January 18, 2011. The LUR model, a crucial component of the ESCAPE project, underwent thorough validation, the details of which can be found at (http://www.escapeproject.eu/)^39^. The model’s performance was assessed using the leave-one-out cross-validation method, and the cross-validation R^2^ for PM_2.5_, PM_10_, NO_2_, and NO_x_ were 77%, 88%, 87%, and 88%, respectively^39^.

Yearly PM_2.5_ and NO_x_ concentration data were only accessible for 2010, while NO_2_ concentration data were accessible for 2005–2007 and 2010. Lastly, PM_10_ information for 2007 and 2010 was accessible. For pollutants for which only yearly concentration data from 2010 were available, the respective baseline exposure variable was directly stated. In the meantime, we used the average of multiple annual concentrations as the exposure variable for pollutants for which multiple years of annual concentration data were available within the baseline period. For PM_10_, for example, the exposure level was determined using the mean annual concentrations in 2007 and 2010.

### Polygenic risk score

In the present study, the PRS was calculated by summing the weighted risk estimate, which corresponded to the β coefficient of 44 single-nucleotide polymorphisms (SNPs) obtained from the reported PD Genome-Wide Association Study (GWAS)^41^. Thompson et al. generated a Standard PRS Set, calculated on all UKB individuals, for 28 diseases and eight quantitative traits by meta-analyzing multiple external GWAS sources^42^. Detailed information on genotyping and quality control can be found in previous studies^43^. We used the weighted calculation method to get the Parkinson’s disease PRS (PD-PRS), as illustrated by the following Eq. (1):

1 $${\rm{PRS}}={\sum }_{i=1}^{n}{\beta }_{i}\times {{SNP}}_{i}$$

Where *β* is per allele log odds ratio (ORs) for PD-associated with *SNP*, which was obtained from the existing GWAS research^42^; *SNP* was documented as 0, 1, and 2 in terms of the number of risk alleles; *n* was the total of selected SNPs in the current study. The ORs were derived from a former GWAS study. For this study, subjects were classified into three categories, namely low (<−0.584), intermediate (−0.584–0.289), and high (>0.289) genetic risk^33–35^. These categories were determined using the PRS values’ tertile distribution as analyzed.

### Covariates

Covariates in this study included age (in years, continuous), sex (i.e., female, male), ethnicity (i.e., White or other), average total household income before tax (i.e., <£18,000, £18,000–£29,999, £30,000–£51,999, £52,000–£100,000, >£100,000), educational level (i.e., college or university degree, others), employment status (i.e., employed or retired, others), smoking status (i.e., current, previous, never), alcohol status (i.e., current, previous, never), and body mass index (BMI, in kg/m^2^). The Townsend deprivation index was computed using the previous national census output regions where the participant’s postcode was determined, with a greater score indicating a greater degree of deprivation socioeconomically^44^. Participants’ demographic attributes (such as birth date, sex, and ethnicity) were acquired from respective NHS Primary Care Trust registries prior to their entry at the assessment center. Touch-screen questionnaires were used to collect sociodemographic data (mean overall household income before taxes, level of education, and occupational status) and behavioral information (alcohol and smoking status) at the baseline assessment center visit^36^. Height and weight were assessed by nurses with sufficient training, and BMI was calculated by the quotient of weight (in kg) and height (in m^2^). We considered the number of long-term morbidities, as this index is associated with lifespan. Detailed definitions can be found in Supplementary Table 8. We included PD-PRS, a genotyping array, and the first ten ancestry principal components as covariates. The missing data proportions for covariates were as follows: 14.4% for household income, less than 1% for employment status, alcohol status, smoking status, and the Townsend Deprivation Index. To address this, we conducted multiple imputations using the fully conditional specification (FCS) method to impute the missing covariate data^45–47^.

### Statistical analyses

Mean ± standard deviation (SD) and number (percentage) were used to represent continuous and categorical variables. Using Cox proportional hazard regression models, the hazard ratio (HR) and 95% confidence interval (CI) for the links between the pollutants of ambient air and genetic risk with incident PD were calculated. The proportionality assumptions were evaluated using Schoenfeld residuals (Supplementary Figs. 3–6). Model 1 utilized a Cox regression model with no adjustments. Model 2 was regulated with respect to sex, age, alcohol consumption, BMI, educational level, smoking status, household income, employment status, and the Townsend deprivation index. Model 3 was modified to take model 2 and the long-term morbidity count into account. Moreover, we performed multiple sensitivity analyses to evaluate the outcomes’ validity. To compare the results of multiple imputations, we first restrict the analysis to subjects with complete data on age, sex, ethnicity, education level, employment status, household income, and Townsend deprivation index covariates. To reduce the impact of reverse causation, we did not include those participants who developed PD during the initial two follow-up years. Third, we made additional adjustments for other covariates (BMI, alcohol consumption, smoking status, the number of long-term morbidities, Parkinson’s disease polygenic risk score, genotyping array, and the first ten principal components of ancestry) to avoid potential confounding. Lastly, we conducted stratified analyses to explore how air pollutants are associated with incident PD, considering age (<65; ≥65), sex (male; female), BMI (high: above median value; low: below median value; median = 26.85), Townsend deprivation index (high: above median value; low: below median value; median = −2.3464), and the count of long-term morbidities (none, one, two or more).

We investigated the dose-response association between pollutants in air and PD risk using restricted cubic spline regressions (knot = 3, degree of freedom = 9). As a reference group, we analyzed the coupled effect of pollutants in air and PD-PRS on the probability of developing PD using subjects in the lowest quartile of air pollution and lowest PRS. Within a 4 × 3 grouping framework, we were able to comprehensively analyze the relationship between air pollutants and genetic-based PD risk. A fully adjusted model with a multiplicative interaction term was used to assess the association between pollutants in air and PRS tertiles. Furthermore, we assessed the association between air pollution and PD by stratifying the data based on PD-PRS tertiles.

All information was analyzed using R (version 4.2.3), and the statistical significance (two-tailed) was established to a P value less than 0.05.

### Reporting summary

Further information on research design is available in the Nature Research Reporting Summary linked to this article.

### Supplementary information

Supplementary Information

Reporting Summary

## Data Availability

The data used in the present study are available from UKB with restrictions applied. Data were used under license and are thus not publicly available. Access to the UKB data can be requested through a standard protocol (https://www.ukbiobank.ac.uk/register-apply/). Data used in this study are available in the UK Biobank under application number 19542. All data supporting the findings described in this manuscript are available in the article and in the Supplementary Information and from the corresponding author upon request.

## Code availability

The code for reproducing the results can be found on [https://github.com/yihezuiji/hymdata/blob/main/airpollution_COX.md].
