# Supplementary material for: Genetic susceptibility modifies the association of long-term air pollution exposure on Parkinson’s disease
Source: NPJ Parkinsons Dis. 2024 Jan 17;10:23. doi: 10.1038/s41531-024-00633-1 (PMC10794179; doi:10.1038/s41531-024-00633-1)

Supplementary Information

Genetic susceptibility modifies the association of long-term air pollution exposure on Parkinson's disease.

Supplementary Table 1. Pearson correlation coefficient matrix for air pollutants

Supplementary Table 2. Association between long-term exposure to air pollution and Parkinson disease (PD) by excluding PD cases occurred in the first 2 years of follow-up.

Supplementary Table 3. Association between long-term exposure to air pollution and Parkinson disease (PD) after additional adjustment for other covariates.

Supplementary Table 4. Association between long-term exposure to air pollution and Parkinson's disease (PD) restricted to participants with complete covariates.

Supplementary Table 5. Association between long-term exposure to air pollution and parkinson's disease (PD) in subgroups stratified.

Supplementary Table 6. Association between genetic risk and Parkinson disease (PD).

Supplementary Table 7. International Classification of Diseases (ICD)-10 codes used to identify parkinson's disease (PD) diagnosis.

Supplementary Table 8. Definition and List of Long-Term Morbidities

Supplementary Figure 1. Exposure-response relationships of each air pollutant with incident Parkinson disease among UK Biobank 312,009 participant

Supplementary Figure 2. Flow of participants through study

Supplementary Figure 3. Schoenfeld residuals test of the Cox proportional hazards assumption based on NO_2 and related covariates.

Supplementary Figure 4. Schoenfeld residuals test of the Cox proportional hazards assumption based on NO_x and related covariates.

Supplementary Figure 5. Schoenfeld residuals test of the Cox proportional hazards assumption based on $PM_{2.5}$ and related covariates.

Supplementary Figure 6. Schoenfeld residuals test of the Cox proportional hazards assumption based on PM_{10} and related covariates.

Supplementary Table 1. Pearson correlation coefficient matrix for air pollutants

	NO_2 (μg/m^3)	NO_x (μg/m^3)	$PM_{2.5}$ (μg/m^3)	$PM_{2.5-10}$ (μg/m^3)	PM_{10} (μg/m^3)
NO_2 (μg/m^3)	1.00				
NO_x (μg/m^3)	0.80*	1.00			
$PM_{2.5}$ (μg/m^3)	0.75*	0.87*	1.00		
$PM_{2.5-10}$ (μg/m^3)	0.23*	0.26*	0.25*	1.00	
PM_{10} (μg/m^3)	0.72*	0.63*	0.62*	0.55*	1.00

*Indicates P <0.001

Supplementary Table 2. Association between long-term exposure to air pollution and Parkinson disease (PD) by excluding PD cases occurred in the first 2 years of follow-up.

Air pollution	HR (95%CI) for Continuous*	Air pollution concentrations (quartile)				P for trend
		Q1	Q2	Q3	Q4	
$PM_{2.5}$						
Model 1	0.792 (0.660-0.951)	1.000 (Ref.)	0.965 (0.866-1.076)	0.946 (0.848-1.056)	0.887 (0.794-0.989)	0.031
Model 2	0.876 (0.699-1.099)	1.000 (Ref.)	1.019 (0.902-1.151)	1.016 (0.897-1.151)	0.987 (0.863-1.128)	0.866
PM_{10}						
Model 1	1.076 (0.874-1.324)	1.000 (Ref.)	1.081 (0.968-1.207)	0.971 (0.869-1.085)	1.051 (0.940-1.176)	0.843
Model 2	1.417 (1.099-1.826)	1.000 (Ref.)	1.144 (1.011-1.295)	1.047 (0.923-1.188)	1.212 (1.061-1.384)	0.029
NO_2						
Model 1	1.018 (0.976-1.063)	1.000 (Ref.)	0.938 (0.839-1.048)	0.992 (0.888-1.107)	1.084 (0.972-1.209)	0.088
Model 2	1.086 (1.027-1.148)	1.000 (Ref.)	0.989 (0.873-1.121)	1.085 (0.957-1.230)	1.284 (1.123-1.469)	<0.001
NO_X						
Model 1	0.960 (0.913-1.010)	1.000 (Ref.)	0.965 (0.866-1.077)	0.979 (0.878-1.092)	0.915 (0.820-1.021)	0.153
Model 2	1.012 (0.953-1.075)	1.000 (Ref.)	1.020 (0.902-1.153)	1.075 (0.949-1.218)	1.055 (0.922-1.207)	0.310

Abbreviations: PD, Parkinson's disease; HR, hazards ratio; CI, confidence interval; $PM_{2.5}$, fine particulate matter with diameter ≤2.5μm; PM_{10}, particulate matter with diameter ≤10μm; NO_2, nitrogen dioxide; NO_X, nitrogen oxides; Ref, reference.

*Hazards ratio (HR) per 5-μg/m^3 change of $PM_{2.5}$ level, per 10-μg/m^3 change of PM_{10} and NO_2 levels and per 20-μg/m^3 change of NO_X level.

P value for trend calculated treating the air pollution concentrations (quartile) as a continuous variable.

Model 1: Unadjusted.

Model 2: Adjusted for age, sex, ethnicity, education level, employment status, household income, and Townsend deprivation index.

Supplementary Table 3. Association between long-term exposure to air pollution and Parkinson disease (PD) after additional adjustment for other covariates.

Air pollution	HR (95%CI) for Continuous*	Air pollution concentrations (quartile)				*P* for trend
		Q1	Q2	Q3	Q4	
$PM_{2.5}$	0.936 (0.751-1.166)	1.00 (Ref.)	1.013 (0.892-1.150)	1.021 (0.898-1.161)	1.012 (0.889-1.152)	0.833
PM_{10}	1.359 (1.055-1.751)	1.00 (Ref.)	1.099 (0.965-1.250)	1.009 (0.885-1.150)	1.201 (1.055-1.366)	0.026
NO_2	1.080 (1.024-1.138)	1.00 (Ref.)	0.987 (0.866-1.125)	1.074 (0.943-1.223)	1.229 (1.082-1.396)	<0.001
NO_X	1.022 (0.963-1.084)	1.00 (Ref.)	0.988 (0.869-1.123)	1.042 (0.916-1.185)	1.061 (0.933-1.206)	0.269

Abbreviations: PD, Parkinson's disease HR, hazards ratio; CI, confidence interval; $PM_{2.5}$, fine particulate matter with diameter ≤2.5μm; PM_{10}, particulate matter with diameter ≤10μm; NO_2, nitrogen dioxide; NO_X, nitrogen oxides; Ref, reference.

*Hazards ratio (HR) per 5-μg/m^3 change of $PM_{2.5}$ level, per 10-μg/m^3 change of PM_{10} and NO_2 levels and per 20-μg/m^3 change of NO_X level.

P value for trend calculated treating the air pollution concentrations (quartile) as a continuous variable.

Multifactorial adjustments were made for age, sex, BMI, alcohol consumption, smoking status, education level, employment status, Townsend deprivation index, and the number of long-term morbidities, Parkinson disease polygenic risk score, genotyping array, and the first 10 principal components of ancestry.

Supplementary Table 4. Association between long-term exposure to air pollution and Parkinson's disease (PD) restricted to participants with complete covariates.

Air pollution	HR (95%CI) for Continuous*	Air pollution concentrations (quartile)				*P* for trend
		Q1	Q2	Q3	Q4	
$PM_{2.5}$	0.876 (0.699-1.099)	1.00 (Ref.)	1.009 (0.894-1.138)	1.011 (0.894-1.144)	0.985 (0.863-1.124)	0.857
PM_{10}	1.417 (1.099-1.826)	1.00 (Ref.)	1.129 (0.999-1.277)	1.040 (0.918-1.178)	1.225 (1.075-1.396)	0.016
NO_2	1.086 (1.027-1.148)	1.00 (Ref.)	0.964 (0.851-1.091)	1.070 (0.945-1.210)	1.245 (1.091-1.422)	<0.001
NO_X	1.012 (0.953-1.075)	1.00 (Ref.)	1.012 (0.897-1.143)	1.077 (0.952-1.218)	1.035 (0.905-1.182)	0.419

Abbreviations: PD, Parkinson's disease; HR, hazards ratio; CI, confidence interval; $PM_{2.5}$, fine particulate matter with diameter ≤2.5μm; PM_{10}, particulate matter with diameter ≤10μm; NO_2, nitrogen dioxide; NO_X, nitrogen oxides; Ref, reference.

*Hazards ratio (HR) per 5-μg/m^3 change of $PM_{2.5}$ level, per 10-μg/m^3 change of PM_{10} and NO_2 levels and per 20-μg/m^3 change of NO_X level.

P value for trend calculated treating the air pollution concentrations (quartile) as a continuous variable.

Multifactorial adjustments were made for age, sex, ethnicity, education level, employment status, household income, and Townsend deprivation index.

Supplementary Table 5. Association between long-term exposure to air pollution and parkinson disease (PD) in subgroups stratified.

	N	$PM_{2.5}$	PM_{10}	NO_2	NO_X
Age					
<65 years	245,761	0.967 (0.918-1.019)	**1.074 (1.019-1.132)**	**1.077 (1.020-1.138)**	1.003 (0.951-1.058)
≥65 years	66,248	0.988 (0.923-1.058)	1.015 (0.947-1.088)	**1.089 (1.016-1.168)**	1.003 (0.936-1.075)
Sex					
Male	166,528	1.011 (0.960-1.065)	**1.089 (1.035-1.146)**	**1.117 (1.059-1.177)**	1.029 (0.977-1.084)
Female	145,481	0.962 (0.895-1.034)	0.986 (0.917-1.059)	1.014 (0.942-1.092)	0.991 (0.922-1.066)
Townsend deprivation index					
Above median value (high deprivation)	155,801	0.982 (0.925-1.042)	1.033 (0.972-1.097)	**1.094 (1.030-1.161)**	1.026 (0.967-1.089)
Below median value (low deprivation)	155,809	0.984 (0.929-1.042)	1.038 (0.980-1.099)	1.036 (0.978-1.097)	1.025 (0.967-1.086)
BMI					
Above median value (high BMI)	155,727	0.989 (0.866-1.129)	1.166 (1.011-1.345)	1.343 (1.049-1.720)	1.104 (0.967-1.260)
Below median value (low BMI)	156,282	1.020 (0.887-1.172	1.116 (0.961-1.296)	1.069 (0.831-1.375)	0.995 (0.866-1.144)
The number of long-term conditions					
None	109008	0.981 (0.897-1.073)	0.996 (0.911-1.089)	1.058 (0.965-1.158)	0.993 (0.907-1.087)
One	99994	1.070 (0.988-1.158)	1.065 (0.985-1.153)	**1.133 (1.046-1.227)**	1.066 (0.985-1.155)
Two or more	109008	0.949 (0.890-1.011)	**1.065 (1.001-1.134)**	1.045 (0.981-1.114)	1.002 (0.940-1.068)

Abbreviations: PD, Parkinson's disease; HR, hazards ratio; CI, confidence interval; $PM_{2.5}$, fine particulate matter with diameter ≤2.5µm; PM_{10}, particulate matter with diameter ≤10µm; NO_2, nitrogen dioxide; NO_X, nitrogen oxides.

P-value for interaction: the interaction between air pollutions with age and sex.

All model was adjusted for age (stratified by sex), sex (stratified by age), BMI, alcohol consumption, smoking status, education level, employment status, Townsend deprivation index, and the number of long-term morbidities.

Supplementary Table 6. Association between genetic risk and Parkinson disease (PD).

Genetic Risk	HR (95%CI) for Continuous*	Low (n = 117,068)	Intermediate (n =117,169)	High (n = 117,678)	*P* for trend
No. of PD cases / person-years		589/894,439	814/892,839	1,206/898,135	
Model 1	1.406 (1.351-1.463)	1.000 (Ref.)	1.390 (1.250-1.546)	2.053 (1.860-2.266)	<0.001
Model 2	1.409 (1.353-1.467)	1.000 (Ref.)	1.441 (1.279-1.624)	2.169 (1.942-2.423)	<0.001
Model 3	1.482 (1.352-1.623)	1.000 (Ref.)	1.442 (1.279-1.624)	2.182 (1.952-2.438)	<0.001

Abbreviations: PD, Parkinson disease; HR, hazards ratio; CI, confidence interval; Ref, reference.

P value for trend calculated treating the genetic risk score as a continuous variable.

Model 1: Unadjusted.

Model 2: Adjusted for age, sex, BMI, alcohol consumption, smoking status, education level, employment status, household income, and Townsend deprivation index.

Model 3: Included model 2 plus Parkinson disease polygenic risk score, genotyping array, the number of long-term morbidities, and the first 10 principal components of ancestry.

Supplementary Table 7. International Classification of Diseases (ICD) - 10 codes used to identify parkinson's disease (PD) diagnosis.

	ICD-9	ICD-10	Self-reported fields
Parkinson's Disease	332.0, 332.1, 333.0	G20, G21, G21.0, G21.1, G21.2, G21.3, G21.4, G21.8, G21.9, G22, G23.0, G23.1, G23.2, G23.3, G23.8, G23.9, G25.9, G26, G90.3	20002 (1262)

Abbreviations: ICD, International Classification of Diseases.

Supplementary Table 8. Definition and List of Long-Term Morbidities

	Morbidity grouping*	Conditions included	Code
1	Hypertension	Hypertension	1065
		Essential hypertension	1072
2	Coronary heart disease	Heart attack/MI	1075
		Angina	1074
3	Diabetes	Diabetic nephropathy	1607
		Diabetic neuropathy/ulcers	1468
		Diabetes	1220
		Type 1 diabetes	1222
		Type 2 diabetes	1223
		Diabetic eye disease	1276
4	Stroke/TIA	Stroke	1081
		TIA	1082
		Subarachnoid haemorrhage	1086
		Brain haemorrhage	1491
		Ischaemic stroke	1583
5	Atrial fibrillation	Atrial fibrillation	1471
6	Heart failure	Cardiomyopathy	1079
		Hypertrophic cardiomyopathy	1588
		Heart failure/pulmonary oedema	1076
7	Peripheral vascular disease	Peripheral vascular disease	1067
		Leg claudication/intermittent claudication	1087
8	COPD	COPD/Chronic obstructive pulmonary disease	1112
		Emphysema/Chronic bronchitis	1113
		Emphysema	1472
9	Asthma	Asthma	1111
10	Bronchiectasis	Bronchiectasis	1114
11	Cancer*	"yes"/"no" to "have you ever had cancer?"	
12	Dyspepsia	Gastro-oesophageal reflux (GORD)	1138
		Oesophagitis/Barrett's oesophagus	1139
		Gastric stomach ulcers	1142
		Gastric erosions/gastritis	1143
		Duodenal ulcer	1457
		Dyspepsia/indigestion	1510
		Hiatus hernia	1474
		Helicobacter pylori	1442
13	Diverticular disease	Diverticular disease/diverticulitis	1458
14	Irritable bowel syndrome	Irritable bowel syndrome	1154
15	Chronic liver disease	Oesophageal varices	1141

		Non infective hepatitis	1157
		Liver failure/cirrhosis	1158
		Primary biliary cirrhosis	1506
16	Inflammatory bowel disease	Inflammatory bowel disease	1461
		Crohn’s disease	1462
		Ulcerative colitis	1463
17	Constipation	Constipation	1599
18	Viral hepatitis	Hepatitis B	1579
		Hepatitis C	1580
		Hepatitis D	1581
19	Depression	Depression	1286
		Postnatal depression	1531
20	Anxiety	Anxiety/panic attacks	1287
		Nervous breakdown	1288
		Post-traumatic stress disorder	1469
		Obsessive compulsive disorder	1615
		Stress	1614
		Insomnia	1616
		Psychological/psychiatric problem	1243
21	Schizophrenia/Bipolar affective disorder	Scizophrenia	1289
	Bipolar	Mania	1291
		Bipolar disorder	1291
		Manic depression	1291
22	Connective tissue diseases	Myositis/myopathy	1322
		Systemic lupus erythematosus/SLE	1381
		Connective tissue disorder	1373
		Sjogren’s syndrome.sicca syndrome	1382
		Dermatopolymyositis	1383
		Scloeroderma/systemic sclerosis	1384
		Rheumatoid arthritis	1464
		Psoriatic arthropathy	1477
		Dermatomyositis	1480
		Polymyositis	1481
23	Painful conditions	Polymyalgia rheumatica	1377
		Back pain	1534
		Joint pain	1537
		Headaches (not migraine)	1436
		Sciatica	1476
		Plantar fasciitis	1540
		Carpal tunnel syndrome	1541
		Fibromyalgia	1542
		Arthritis	1538

		Shingles	1573
		Disc problem	1532
		Prolapsed disc/slipped disc	1312
		Spine arthritis/spondylitis	1311
		Ankylosing spondylitis	1313
		Back problem	1294
		Osteoarthritis	1465
		Gout	1466
		Cervical spondylosis	1478
		Trigeminal neuralgia	1523
		Disc degeneration	1533
		Trapped nerve/compressed nerve	1257
24	Osteoporosis	Osteoporosis	1309
		Thyroid problem (not cancer	1224
		Hyperthroidism/thyrotoxicosis	1225
		Hypothyroidism/myxoedema	1226
25	Thyroid disorders	Grave's disease	1522
		Thyroid goitre	1610
		Thyroitis	1428
		Alcohol dependency	1408
26	Alcohol problems	Alcoholic liver disease/alcoholic cirrhosis	1604
		Polycystic kidney	1427
		Diabetic nephropathy	1607
		Renal/kidney failure	1192
27	Chronic kidney disease	Renal failure requiring dialysis	1193
		Renal failure not requiring dialysis	1194
		Kidney nephropathy	1519
		Immunoglobulin A (IgA) nephropathy	1520
		Prostate problem (not cancer)	1207
28	Prostate disorders	Enlarged prostate	1396
		Benign prostatic hypertrophy	1516
29	Glaucoma	Glaucoma	1277
30	Epilepsy	Epilepsy	1264
31	Dementia	Dementia/Alzheimer/cognitive impairment	1263
32	Psoriasis or eczema	Eczema/dermatitis	1452
		Psoriasis	1453
33	Migraine	Migraine	1265
34	Chronic sinusitis	Chronic sinusitis	1416
35	Anorexia or bulimia	Anorexia, bulimia/other eating disorder	1470
36	Parkinson's disease	Parkinson's disease	1262
37	Multiple sclerosis	Multiple sclerosis	1261

38	Chronic fatigue syndrome	Chronic fatigue syndrome	1482
39	Endometriosis	Endometriosis	1402
40	Meniere disease	Meniere disease	1421
41	Pernicious anaemia	Pernicious anaemia	1331
42	Polycystic ovaries	Polycystic ovaries	1350

* Self-report lifetime diagnosis by doctor recorded by nurse-led interview (UK Biobank data field ID: 20002), except cancer diagnosis which was reported by touch-screen questionnaire. The list of disease groupings was based on Barnett et al (2012). In the present study, we did not include patients with Parkinson's disease (code 1262) and dementia (code 1263) at baseline.

Supplementary Figure 1. Exposure-response relationships of each air pollutant with incident Parkinson disease among UK Biobank 312,009 participant (knot = 3, degree of freedom = 9)

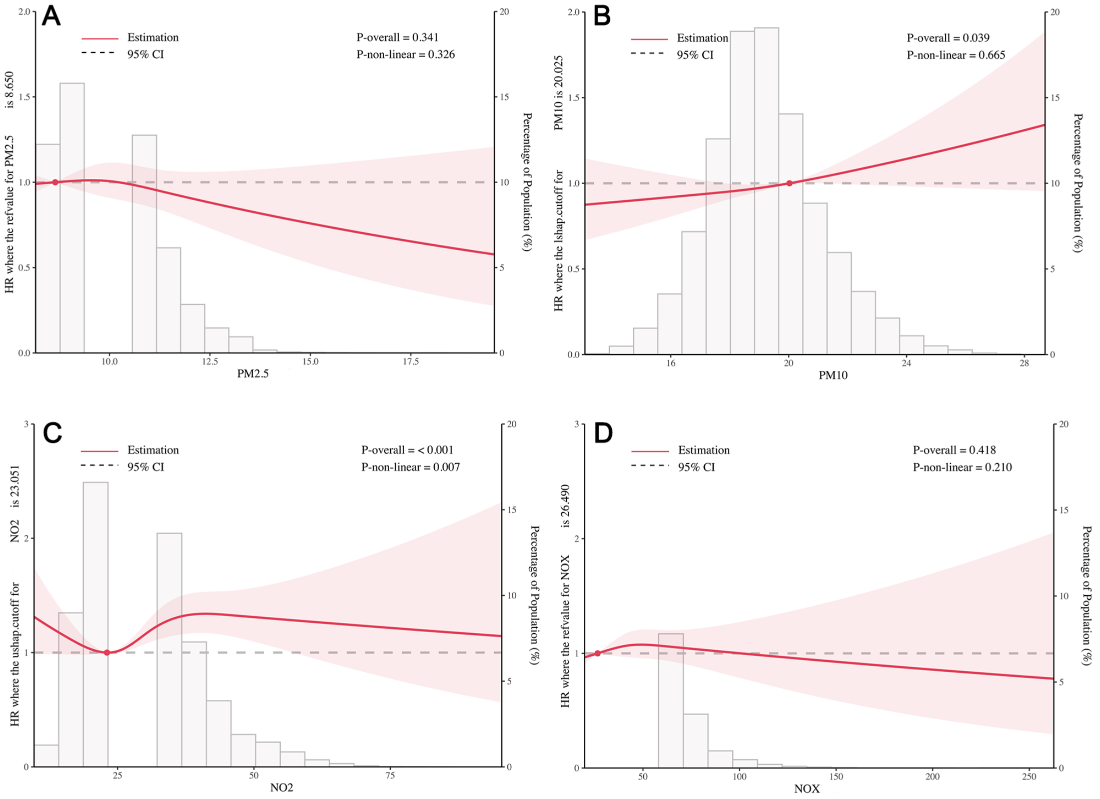

The exposure-response relationship of concentrations for each air pollutants with hazard ratios for incident PD was assessed using restricted cubic spline analysis with 3 knots.

Both were adjusted for age, sex, BMI, education level, employment status, household income, and Townsend deprivation index.

Supplementary Figure 2. Flow of participants through study

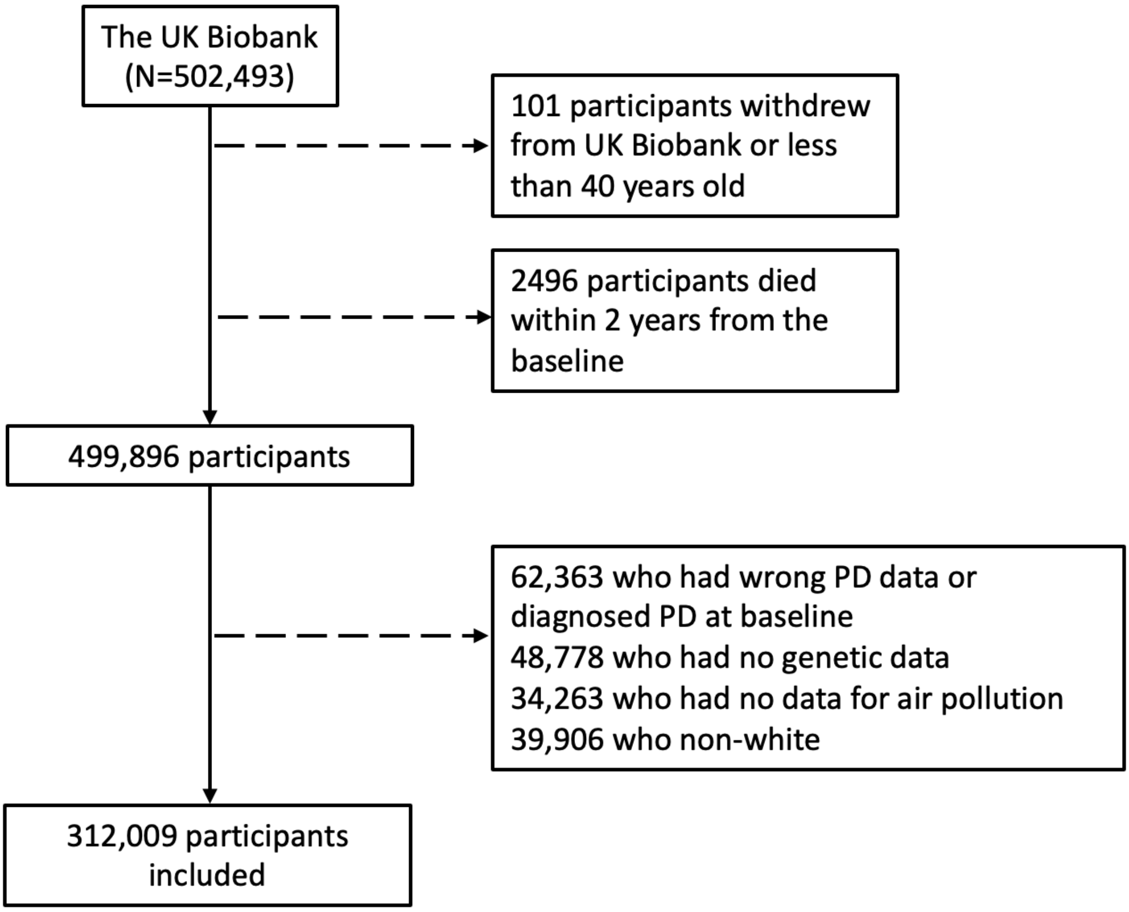

Supplementary Figure 3. Schoenfeld residuals test of the Cox proportional hazards assumption based on NO_2 and related covariates.

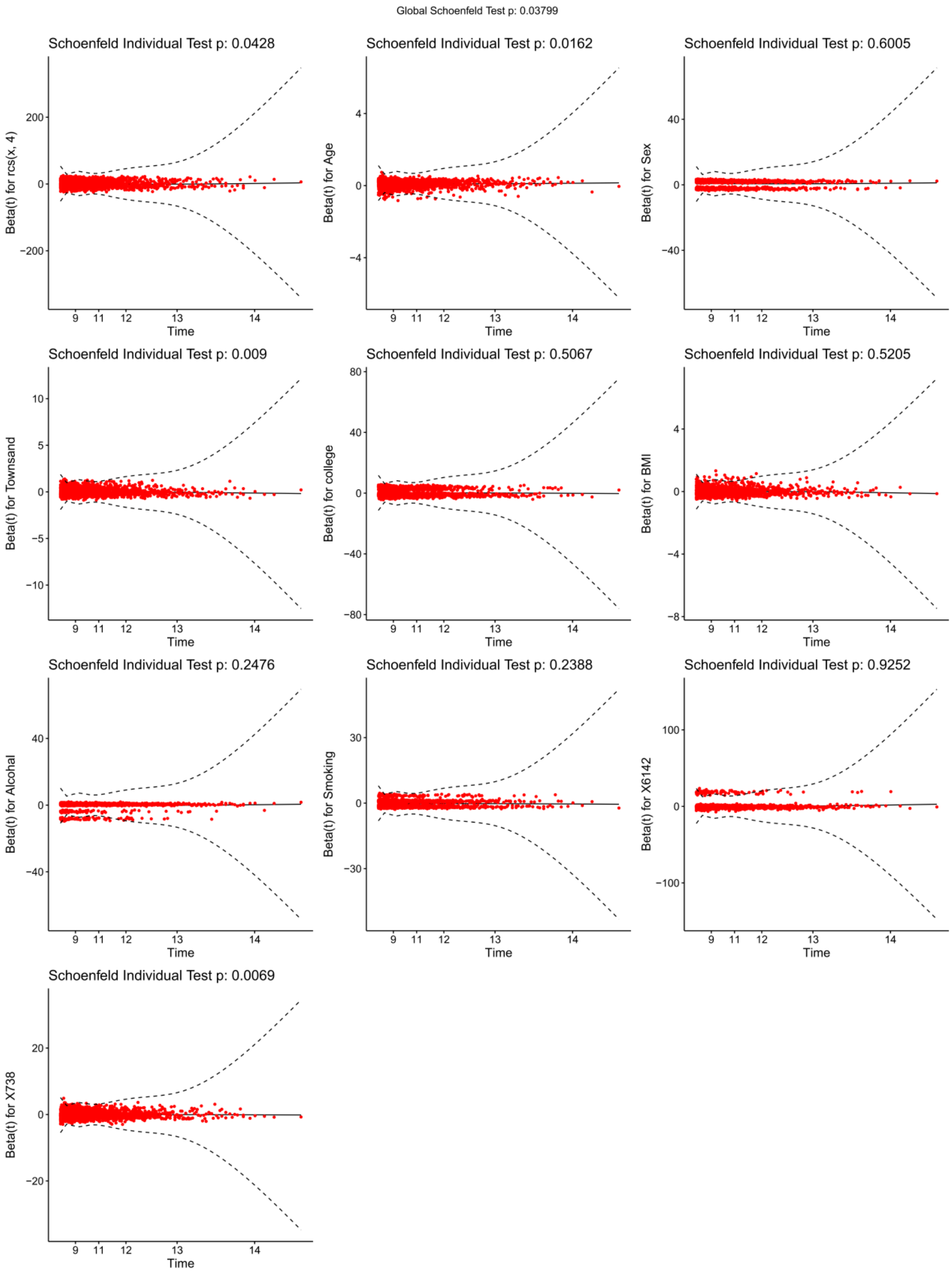

Supplementary Figure 4. Schoenfeld residuals test of the Cox proportional hazards assumption based on NO_x and related covariates.

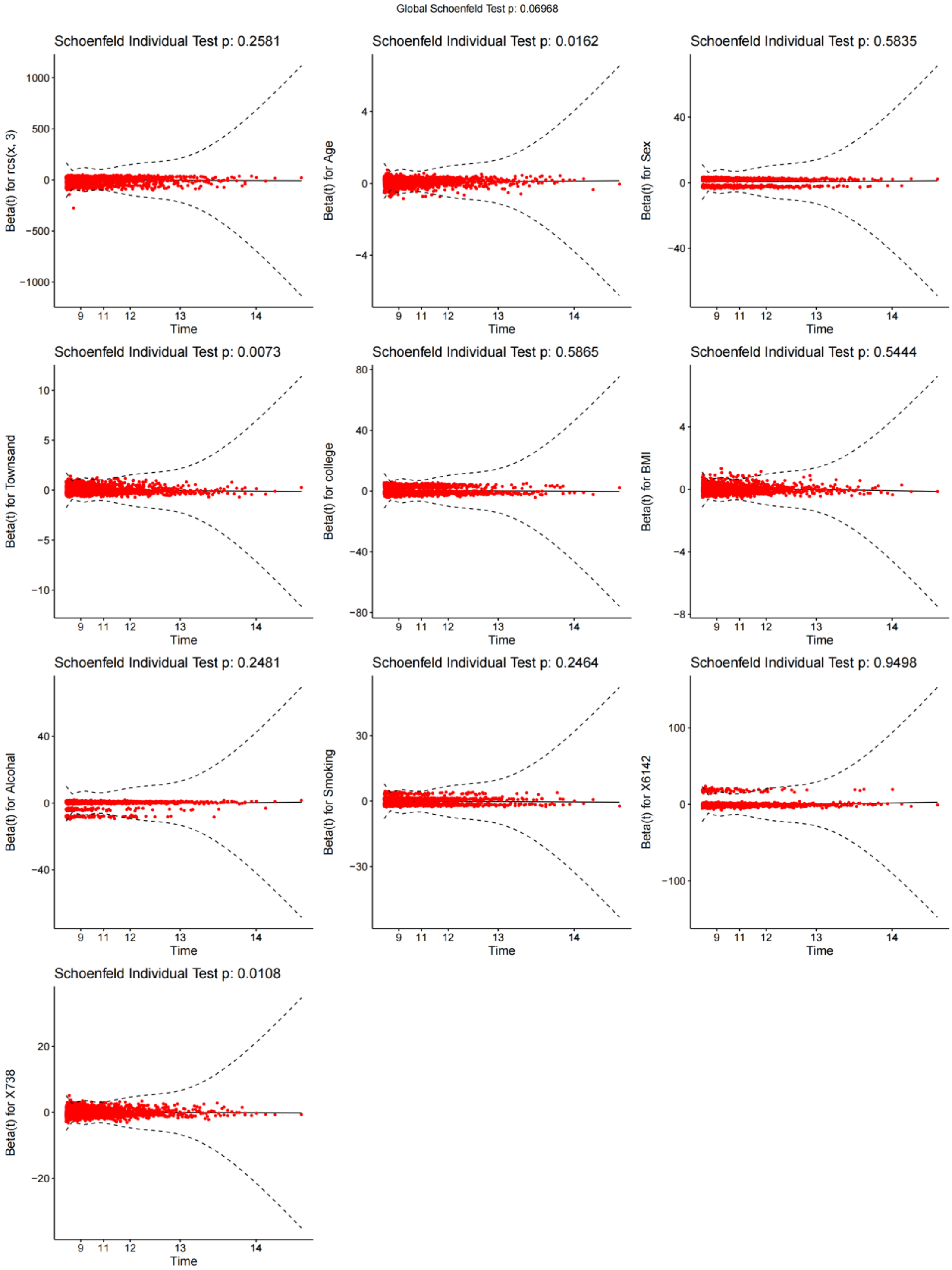

Supplementary Figure 5. Schoenfeld residuals test of the Cox proportional hazards assumption based on $PM_{2.5}$ and related covariates.

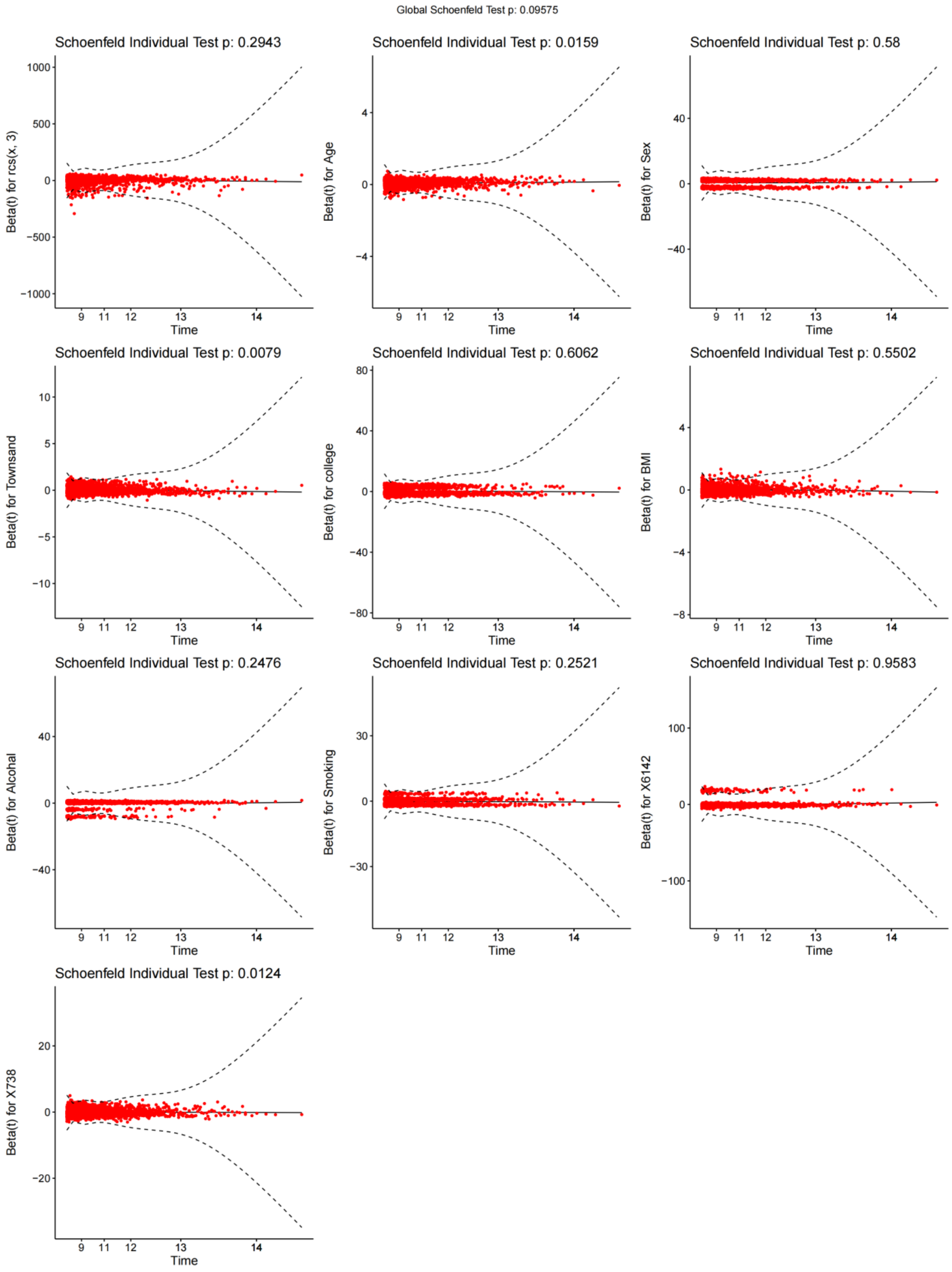

Supplementary Figure 6. Schoenfeld residuals test of the Cox proportional hazards assumption based on PM_{10} and related covariates.

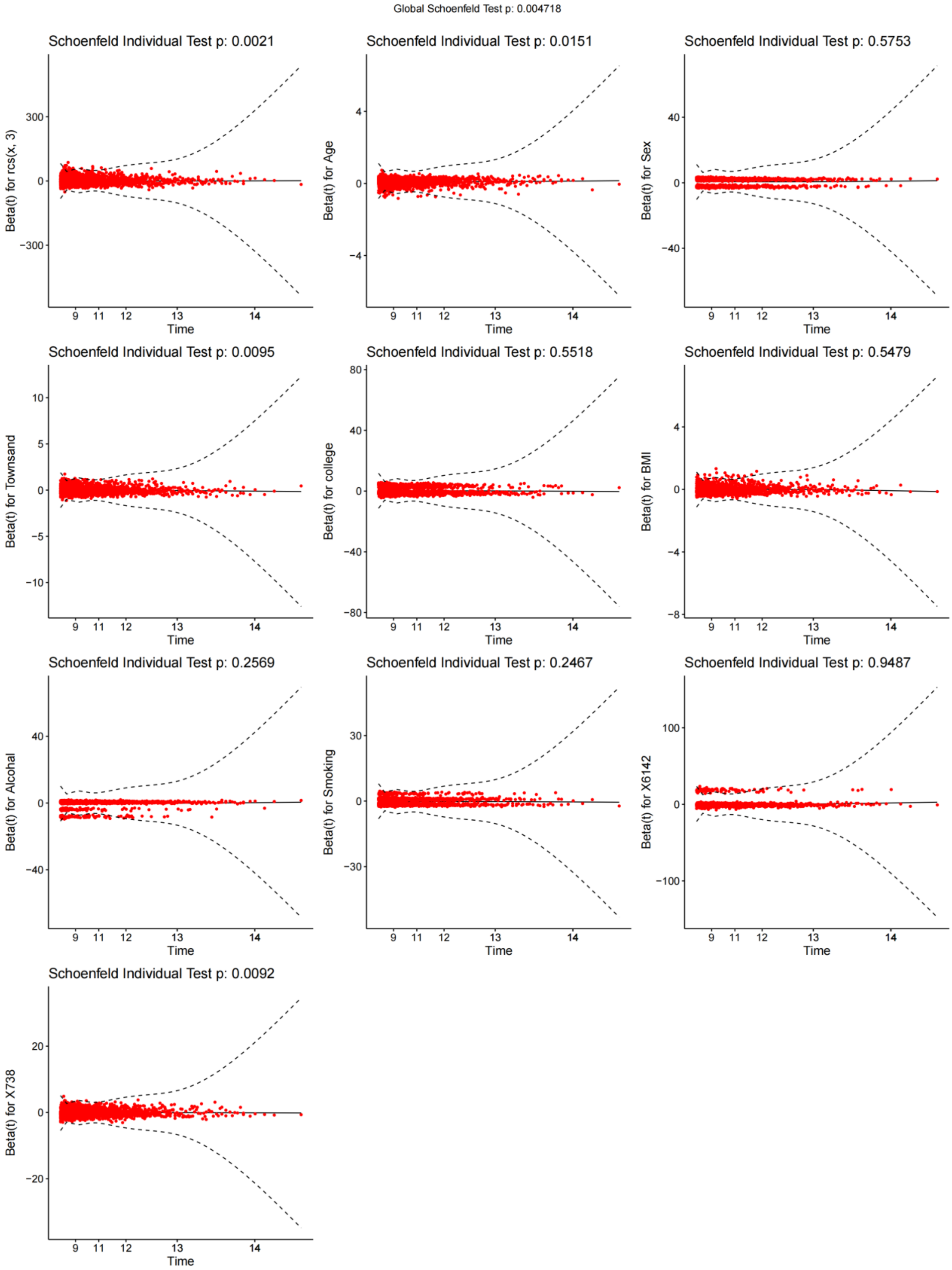

Supplement: Supplementary file 1 — Supplementary Information [file 41531_2024_633_MOESM1_ESM.pdf]
