# Supplementary material for: Genetic susceptibility modifies the association of long-term air pollution exposure on Parkinson’s disease
Source: NPJ Parkinsons Dis. 2024 Jan 17;10:23. doi: 10.1038/s41531-024-00633-1 (PMC10794179; doi:10.1038/s41531-024-00633-1)
Supplement: Supplementary file 2 — Reporting Summary [file 41531_2024_633_MOESM2_ESM.pdf]

nature portfolio

Corresponding author(s): Jin-Tai Yu; Lan Tan

Last updated by author(s): Jan 1, 2024

Reporting Summary

Nature Portfolio wishes to improve the reproducibility of the work that we publish. This form provides structure for consistency and transparency in reporting. For further information on Nature Portfolio policies, see our Editorial Policies and the Editorial Policy Checklist.

Statistics

For all statistical analyses, confirm that the following items are present in the figure legend, table legend, main text, or Methods section.

n/a	Confirmed	
☐	☒	The exact sample size (*n*) for each experimental group/condition, given as a discrete number and unit of measurement
☐	☒	A statement on whether measurements were taken from distinct samples or whether the same sample was measured repeatedly
☐	☒	The statistical test(s) used AND whether they are one- or two-sided<br>*Only common tests should be described solely by name; describe more complex techniques in the Methods section.*
☐	☒	A description of all covariates tested
☐	☒	A description of any assumptions or corrections, such as tests of normality and adjustment for multiple comparisons
☐	☒	A full description of the statistical parameters including central tendency (e.g. means) or other basic estimates (e.g. regression coefficient) AND variation (e.g. standard deviation) or associated estimates of uncertainty (e.g. confidence intervals)
☐	☒	For null hypothesis testing, the test statistic (e.g. *F*, *t*, *r*) with confidence intervals, effect sizes, degrees of freedom and *P* value noted<br>*Give P values as exact values whenever suitable.*
☒	☐	For Bayesian analysis, information on the choice of priors and Markov chain Monte Carlo settings
☒	☐	For hierarchical and complex designs, identification of the appropriate level for tests and full reporting of outcomes
☒	☐	Estimates of effect sizes (e.g. Cohen's *d*, Pearson's *r*), indicating how they were calculated

Our web collection on statistics for biologists contains articles on many of the points above.

Software and code

Policy information about availability of computer code

Data collection	No software used during data collection, and data can be directly download after approval of access application.
Data analysis	R 4.2.3

For manuscripts utilizing custom algorithms or software that are central to the research but not yet described in published literature, software must be made available to editors and reviewers. We strongly encourage code deposition in a community repository (e.g. GitHub). See the Nature Portfolio guidelines for submitting code & software for further information.

Data

Policy information about availability of data

All manuscripts must include a data availability statement. This statement should provide the following information, where applicable:

- Accession codes, unique identifiers, or web links for publicly available datasets
- A description of any restrictions on data availability
- For clinical datasets or third party data, please ensure that the statement adheres to our policy

The data used in the present study are available from UKB with restrictions applied. Data were used under license and are thus not publicly available. Access to the UKB data can be requested through a standard protocol (https://www.ukbiobank.ac.uk/register-apply/). Data used in this study are available in the UK under application number 19542.

Research involving human participants, their data, or biological material

Policy information about studies with human participants or human data. See also policy information about sex, gender (identity/presentation), and sexual orientation and race, ethnicity and racism.

Reporting on sex and gender	Sex (biological) has been included in our analysis which was self-reported during UK Biobank recruitment. Gender has not been collected and thus does not appear in our analysis.
Reporting on race, ethnicity, or other socially relevant groupings	Ethnicity has been included in our analysis which was self-reported during UK Biobank recruitment. Social economic status was measured using Townsend deprivation index calculated immediately prior to participant joining UK Biobank. It was calculated based on the preceding national census output areas. Each participant is assigned a score corresponding to the output area in which their postcode is located. No information of sex and ethnicity was involved in the calculation of social economic status. The ethnicity and social economic status was leveraged as covariates in our analysis as has been previously reported as important risk factors to specific diseases and been adopted to established clinical risk scales.
Population characteristics	This study used 312,009 participants without Parkinson's disease (PD) at baseline who had a median age of 57 years (standard deviation (SD) 8.01), 53% of whom were female and all of whom were white. There were 91,618 (29%) participants with a college or university degree, a body mass index of 27.6 (SD 4.8), and 32,120 (10.6%) current smokers. Median follow-up to March 2023 was 12.07 (range: 10.46-15.01) years, with 2,356 developing PD (15.76%).
Recruitment	The UKB enrolled the participants aged 40-69 years between 2006 and 2010 for baseline assessments in 22 centers across the UK. The assessment visits included comprehensive range of data collections, covering interviews, physical measures, biological samples, imaging, and genotyping. The database is linked to national health datasets, including primary care, hospital inpatient, death, and cancer registration data. The UK Biobank diligently collected estimates of ambient air pollutants, including PM2.5, PM10, NO2, and NOx, spanning the years 2005 to 2007 and specifically focusing on the year 2010.
Ethics oversight	UK Biobank has received ethical approval from the North West Multi-centre Research Ethics Committee (MREC, https://www.ukbiobank.ac.uk/learn-more-about-uk-biobank/about-us/ethics), and informed consent through electronic signature was obtained from study participants. This study utilized the UK Biobank Resource under application number 19542.

Note that full information on the approval of the study protocol must also be provided in the manuscript.

Field-specific reporting

Please select the one below that is the best fit for your research. If you are not sure, read the appropriate sections before making your selection.

☒ Life sciences ☐ Behavioural & social sciences ☐ Ecological, evolutionary & environmental sciences

For a reference copy of the document with all sections, see nature.com/documents/nr-reporting-summary-flat.pdf

Life sciences study design

All studies must disclose on these points even when the disclosure is negative.

Sample size	The UK Biobank contains data on more than 500,000 participants, of whom 312,009 did not have Parkinson's disease at baseline and had complete polygenic risk scores and air pollution-related data. As the focus of this study was on the role of genetic susceptibility as a modifier between air pollution and Parkinson's disease, the sample size of this study was adequate for statistical analysis as we had a larger sample size than previous studies conducted in related research areas.
Data exclusions	This investigation precluded (N = 64,940) persons with a history of baseline PD and those with confirmed PD based on medical data. Participants with incomplete genetic information (N = 48,778) and air pollution information (N = 34,263) were therefore not included. Additionally, we excluded individuals of non-White descent (self-reported by participants; n = 39,906), as the genetic instrument used in this study was constructed based on data from White participants.
Replication	Our data analysis was conducted on a comprehensive dataset, providing details on the data collection process, time frame, and sample size. The statistical models used for analysis were explicitly outlined to ensure transparency. To ensure data reproducibility, we implemented rigorous measures during data cleaning and outlier handling, following standardized procedures throughout the data processing pipeline.
Randomization	Not relevant for this study
Blinding	This study did not require blinding as no subjective evaluation by on observer was involved.

Reporting for specific materials, systems and methods

We require information from authors about some types of materials, experimental systems and methods used in many studies. Here, indicate whether each material, system or method listed is relevant to your study. If you are not sure if a list item applies to your research, read the appropriate section before selecting a response.

Materials & experimental systems

n/a	Involved in the study
☒	☐ Antibodies
☒	☐ Eukaryotic cell lines
☒	☐ Palaeontology and archaeology
☒	☐ Animals and other organisms
☒	☐ Clinical data
☒	☐ Dual use research of concern
☒	☐ Plants

Methods

n/a	Involved in the study
☒	☐ ChIP-seq
☒	☐ Flow cytometry
☒	☐ MRI-based neuroimaging

Plants

Seed stocks	*Report on the source of all seed stocks or other plant material used. If applicable, state the seed stock centre and catalogue number. If plant specimens were collected from the field, describe the collection location, date and sampling procedures.*
Novel plant genotypes	*Describe the methods by which all novel plant genotypes were produced. This includes those generated by transgenic approaches, gene editing, chemical/radiation-based mutagenesis and hybridization. For transgenic lines, describe the transformation method, the number of independent lines analyzed and the generation upon which experiments were performed. For gene-edited lines, describe the editor used, the endogenous sequence targeted for editing, the targeting guide RNA sequence (if applicable) and how the editor was applied.*
Authentication	*Describe any authentication procedures for each seed stock used or novel genotype generated. Describe any experiments used to assess the effect of a mutation and, where applicable, how potential secondary effects (e.g. second site T-DNA insertions, mosiacism, off-target gene editing) were examined.*
